# The Production of Diphtheria Antitoxin

**Published:** 1895-04

**Authors:** H. D. Gill


### II. The Production of Diphtheria Antitoxin.

BY H. D. GILL, V.S.

Out of seventy-five horses I have had under my charge three-fourths are either balkers, kickers, runaways, or have some habit which renders them unfit or unsafe for general use. On account of these faults I have been able to get young and healthy horses for a very low figure.

Coarse-bred horses between the ages of four and six, well muscled and nourished, and having had regular work up to the time of inoculation, are the ones selected by me as best fitted for this purpose.

Green horses, or those of a nervous temperament, seem to be physically unable to withstand even small doses of toxin, which in them causes very sudden and alarming nervous symptoms, from which they may die or very slowly recover after injections, and therefore are rendered more susceptible to the poison. It is a waste of time and money to continue with such subjects.

The circulation, respiration and temperature must be normal, they must be free from any disease and stand the mallein test for glanders.

Lameness insufficient to cause systemic disturbance or interfere with daily exercise should not be a bar. No objection to color. (*See further on.*) Clipping and thorough grooming improve the general condition of the animal, and the skin is more easily rendered aseptic.

The food should be varied, but of the best, the amount being governed by the general condition, weight and exercise. I have noticed that in horses receiving regular diet and little or no exercise the reaction to the toxin is considerably increased, causing a loss of valuable time to allow for convalescence.

As a rule horses, particularly those with swellings, do best in box stalls, and all but those exhibiting marked nervous symptoms should be loose; the latter should be kept perfectly quiet in single stalls with no food for at least one day, and then with only half the regular allowance until their return to a perfectly normal condition. With the exception of the last mentioned they should be exercised daily, stiff ones as well, for I have found that forced exercise to these causes a more rapid absorption of the infiltration, and a general improvement follows.

For *inoculation*, two sterilized hyperdermatic syringes are used, one holding ten cubic centimetres and the other holding about sixty cubic centimetres. The first for small and the latter for large doses.

Syringes with asbestos plungers are preferable, because they can be sterilized by heat without altering or destroying their usefulness. Syringes with plungers of other material are made sterile by soaking for twenty-four hours in 5 per cent. solution of carbolic acid.

The flank, brisket and the upper and middle third of the thorax posterior to scapula are the points generally selected for inoculation.

Injections are not made in the neck, because the subsequent issue-change interferes more or less with the isolation of the jugular vein when we come to bleed; nor in the extremities, as the subsequent swelling causes painful and limited movement of the limb.

Within a radius of at least six inches from the point of inoculation the hair should be either closely clipped or shaved, the part washed with soap and water, followed by either a 5 per cent. solution of carbolic acid, or a solution of $\frac{1}{800}$ bichloride of mercury.

The proper amount of toxin solution is drawn into the syringe, all air being expelled; the needle is dipped into the carbolic solution and then very quickly forced obliquely through and under the skin for at least two inches. This prevents the escape of the toxin through the puncture made, particularly when any considerable amount is injected. Great care must be exercised not to inject air with the toxin.

Toxin solutions, unless filtered, should stand for some time before being used, so as to allow the dead diphtheria bacilli to precipitate, and no more than fifty cubic centimetres should be injected at one point.

The best results have been obtained by commencing with very small doses (one-half cubic centimetre) of weak toxin, repeating with an increased amount not sooner than forty-eight hours, and only then if the temperature is normal and the animal apparently healthy. This is very necessary, as we very often have a secondary reaction.

In a number of animals having tissue enlargements the part affected was aggravated after injection, and terminated either in absorption to a greater or less degree or in suppuration.

At the point of injection, in nearly all cases, a very hard and intensely sensitive swelling is produced, varying in size; when not absorbed it terminates in either abscess, new connective tissue or slough, these conditions being treated in the usual way.

In a number of horses, principally gray, there is developed urticaria; in others the skin becomes hot and tense. When a horse is able to take one hundred to two hundred cubic centimetres of a virulent toxin solution without producing any marked abnormal symptoms it has acquired considerable immunity. A small amount of blood, about one-half litre, is then drawn and tested to determine the amount of antitoxin it contains.

If up to the standard about six litres of blood are drawn from a horse of average size, the injection of toxin is continued for three or four weeks, and then, if conditions are favorable, the bleeding process is repeated, and the strength determined as before. For one week prior to bleeding the horse must be free from all toxic symptoms, during which time it receives no more injections. The horses so bled appear to suffer no inconvenience from the same, but on the contrary are more lively and have an increased appetite. The amount of toxin an animal receives is no indication of the strength of the antitoxin obtained.

Therefore it is necessary that the serum from each and every drawing should be tested.

The horse's susceptibility is determined by symptoms, particularly the temperature, which is taken at 8 A.M. and 7 P.M., daily.

The following is our method for the extraction of blood from a horse: The horse is prepared by shaving and washing as before, and all instruments sterilized by boiling in a 1 per cent. carbonate of soda solution. An incision of about one inch in length is made through the skin with a scalpel over and parallel to the jugular vein. The trocar used is a piece of steel tubing three-eighths of an inch in diameter, and about four inches long; it has thin walls and a sharp beveled point at one end; the sharp point is plunged into the jugular vein, which is distended by an assistant compressing the same below.

To the blunt end of the canula is attached a piece of rubber tubing eighteen inches long, the distal end fitting over a glass tube about four inches in length. Before entering the vein the glass end is put into a flask previously sterilized. The blood flows until the desired amount is obtained. The current is stopped by compressing the rubber tube, and the glass tube is withdrawn. If more blood is to be drawn, this process is repeated. The canula is withdrawn and the wound sterilized, sutured and covered with iodoform and salicylic acid, when healing generally takes place by first intention.

The first drawing is tested, and if found of sufficient strength five or six litres may be extracted. The serum of this blood is used as an immunizing and curative agent.

The flasks used for this purpose are ordinary "Erlenmeyer" and hold from one-half to one litre; when filled they are placed in a refrigerator, where they remain for at least forty-eight hours. By this time the greater amount of the serum has separated from the clot, and is drawn off through a sterilized pipette into bottles holding about twenty-five cubic centimetres. A small piece of camphor is added, the bottles labeled and placed in stock ready for use.

---

The State Veterinary Sanitary Board of Pennsylvania commences its work on June 1, 1895.

